# Application of Rapid Detection Technology for the Determination of γ-Hydroxybutyric Acid

**DOI:** 10.3390/bios16050288

**Published:** 2026-05-15

**Authors:** Nan Li, Xingliang Liu, Boyuan Shi, Chunhui Song, Teng Zhang, Xin Yan, Yingying Li, Xinyi Li, Jun Ma

**Affiliations:** 1 Beijing Key Laboratory of Psychoactive Substances Detection and Control, Beijing Narcotics Control Technology Center, Beijing 100164, China; linanzzzz@126.com (N.L.); liuxingliang0721@hotmail.com (X.L.); hy5049@126.com (B.S.); songchunhui2023@163.com (C.S.); zhangteng_zt2019@163.com (T.Z.); yanxinbucm@126.com (X.Y.); liyy990222@163.com (Y.L.); lixinyi_9806@sina.com (X.L.); 2 National Narcotics Laboratory Beijing Regional Center, Beijing 100164, China

**Keywords:** γ-hydroxybutyric acid, rapid detection, colorimetric method, review

## Abstract

The abuse of γ-hydroxybutyric acid (GHB) and its precursors, γ-butyrolactone (GBL) and 1,4-butanediol (1,4-BD), has increased in recent years, with these substances frequently being illicitly added to beverages. GHB is colorless and odorless and exhibits anesthetic and hypnotic psychoactive effects, which are often exploited in drug-facilitated sexual assault, posing a significant public safety concern. Chromatography–tandem mass spectrometry is a conventional analytical approach for narcotic drug determination due to its high sensitivity and accuracy; however, its large instrumentation footprint and high operational cost limit its suitability for on-site rapid screening. In response to the growing demand for field-deployable analytical tools, rapid detection technologies for GHB have progressively evolved. This review summarizes and compares the advantages and limitations of current rapid detection methods for GHB and discusses their potential future developmental trends, with the aim of providing a reference for researchers and relevant authorities.

## 1. Introduction

Gamma-hydroxybutyric acid (GHB), also known as “liquid ecstasy” or “G water,” is a central nervous system depressant that is clinically used as an anesthetic and exhibits sedative and hypnotic psychoactive effects [1,2]. In recent years, the global prevalence of GHB abuse has increased. Excessive consumption can lead to severe adverse effects, including coma and memory loss, and in extreme cases may result in psychiatric disorders [3]. Moreover, due to its colorless and odorless properties, GHB has been misused as a drug to facilitate sexual assault, posing significant social and public health concerns [4,5,6]. Currently, GHB is regulated in most countries. In China, it was classified as a Class II psychoactive substance in May 2001 and subsequently reclassified as a Class I controlled psychoactive substance in 2007 [7].

**Figure 1:**
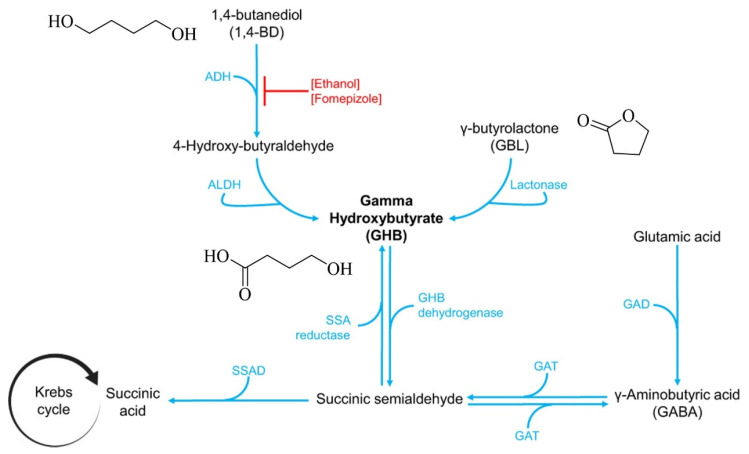
Synthesis and metabolic pathways of GHB [8].

1,4-Butyrolactone (GBL) and 1,4-butanediol (1,4-BD) are precursor substances used in the synthesis of GHB, and can be rapidly metabolized into GHB in vivo, thereby producing similar psychoactive effects (Figure 1) [5,8,9]. In recent years, GBL and 1,4-BD have been illicitly added to beverages as substitutes for GHB and subsequently abused [10,11]. For instance, a beverage known as “Kava Tonic,” which has been widely abused in China, primarily contains GHB and GBL as its active ingredients. However, due to their extensive industrial applications, GBL and 1,4-BD are not currently classified as internationally controlled psychoactive narcotics. On 16 August 2021, China included GBL in the regulatory framework of precursor chemicals used in drug manufacturing, whereas 1,4-BD remains unregulated [12].

Mass spectrometry is currently the most widely used method for analyzing GHB and its precursor substances, owing to its high analytical sensitivity and low detection limits. In 2018, the State Administration for Market Regulation of China issued an industry standard entitled (BJS 201803) “Determination of γ-Butyrolactone and Related Substances in Beverages,” which adopts liquid chromatography–mass spectrometry (LC-MS) to determine GHB, GBL, and 1,4-BD in beverages, with limits of detection and quantification of 1.0 and 2.5 mg kg^−1^, respectively [13]. However, during LC-MS detection, the presence of a memory effect means that excessive injection volumes or high analyte concentrations can lead to residual contamination [14]. In routine practice, samples often require dilution, and complex pretreatment procedures may increase analytical errors. In addition, under acidic conditions, GHB and GBL readily undergo interconversion, potentially altering the original proportions of GHB, GBL, and 1,4-BD in the sample [2].

Gas chromatography–mass spectrometry (GC-MS) is a widely utilized analytical technique in the field of drug detection. However, during analysis, GHB can readily convert to GBL through intramolecular esterification at the high temperatures of the GC injection port; thus, a derivatization step is imperative [14,15]. To ensure analytical accuracy and complete derivatization, the reaction typically requires an extended period, rendering sample preparation complex and time-consuming.

GC-MS and LC-MS are commonly employed for the detection of GHB. However, these techniques rely on large and sophisticated instrumentation, which can impose significant limitations in practical forensic and field applications [14].

Furthermore, GHB exhibits a rapid onset of action following oral administration, typically taking effect within 15 to 30 min, and its effects persist for approximately 6 to 8 h due to rapid metabolism [16,17]. The brief detection window poses significant challenges for accurately measuring GHB concentrations in human biological specimens [18,19]. Consequently, delayed sample collection could lead to lower measured concentrations than the actual amounts consumed, potentially leading to underestimation of its prevalence in case analyses [20,21]. Hence, the development of rapid and reliable detection methods for GHB and its precursors is of considerable importance. Such efforts are vital for effective monitoring of substance abuse, timely identification and prevention of drug-facilitated sexual assault, and the protection of public safety and social stability.

This article reviews current rapid detection methods for GHB, analyzing their respective advantages and disadvantages, and discusses potential future directions in the development of rapid detection technologies. The aim is to provide a valuable reference for relevant regulatory authorities and researchers.

## 2. Methods

### 2.1. Colorimetric Method

Colorimetry, a method employed since the 19th century for identifying plant alkaloids, has become a prevalent technique used in rapid drug detection, particularly following the discovery of colorimetric reactions for compounds such as ephedrine and cocaine [22]. This technique relies on the generation of color or luminescence when the target compounds interact with specific chemical reagents, with the intensity of the resulting signal reflecting the concentration of the analyte. When combined with ultraviolet spectroscopy, colorimetry can also enable quantitative analysis.

#### 2.1.1. General Chemical Colorimetric Method

Alston et al. [23] established a rapid colorimetric screening method for the detection of GHB in human urine. The assay can be completed within 5 min, achieving a detection limit of 0.5 mg mL^−1^ when 0.3 mL of urine is used and 0.1 mg mL^−1^ when the sample volume is increased to 1 mL. However, this method employs concentrated sulfuric acid for the esterification step in the colorimetric reaction. The addition of concentrated sulfuric acid generates substantial heat, which may lead to localized boiling and even splashing, thereby posing safety risks. Its high viscosity also hinders accurate volumetric control. Moreover, excessive use of concentrated sulfuric acid necessitates the addition of large volumes of NaOH in the subsequent step to adjust the pH, leading to sample dilution and a consequent reduction in detection sensitivity. Zhang Shaoyu et al. [24] improved Alston’s method by substituting concentrated sulfuric acid with 6 mol L^−1^ sulfuric acid, enabling a safer and more stable esterification reaction within 5 to 10 min. This modified approach demonstrates improved reproducibility. Notably, even without water-bath heating, allowing the mixture to stand for 5 min produces a comparable, albeit slightly less intense, color change, making it suitable for field applications. The detection limit for GHB in beverages ranges from 0.5 to 2 mg mL^−1^. In urine samples, the detection limit is approximately 0.5 mg mL^−1^.

#### 2.1.2. Enzymatic Assay

Complementing these approaches, an alternative colorimetric method relies on an enzymatic reaction catalyzed by recombinant GHB dehydrogenase (GHBDH). This enzyme catalyzes the oxidation of GHB to succinic semialdehyde (SSA) while concurrently reducing NAD^+^ to NADH. Monitoring the formation of NADH at 340 nm enables both qualitative and quantitative detection of GHB. This technique, also known as the enzymatic assay, is suitable for rapid analysis of biological samples such as blood and urine [25,26]. However, Hasan et al. [27] reported that turbid blood samples and dark-colored urine may interfere with NADH detection, potentially leading to false positive outcomes.

#### 2.1.3. Novel Materials and Sensors Applied in the Colorimetric Method

In recent years, the expanding use of advanced chemical and biological materials in catalysis and electrochemical sensing has facilitated their application in colorimetric detection of GHB [28]. These materials not only accelerate reaction kinetics and amplify analytical signals but also improve specificity and lower detection limits. Gold nanoparticles (AuNPs), owing to their distinctive surface properties and high density of free electrons, exhibit surface plasmon resonance (SPR) upon light irradiation, producing a characteristic absorption peak in the UV-vis spectrum [29]. In this context, NADH produced during the aforementioned GHBDH-catalyzed enzymatic reaction acts as a reducing agent, facilitating the reduction of gold (III) complexes to generate AuNPs. Based on this principle, Hu et al. [30] conducted a pioneering study on the GHB-induced synthesis of AuNPs. Their findings revealed that AuNP formation was characterized by a pronounced absorption peak at 539 nm, accompanied by a visible color change from colorless to pink. Furthermore, the absorption at 539 nm increased linearly with GHB concentrations spanning from 4 to 16 mmol L^−1^. Rodriguez-Nuevalos et al. [31] refined this method by introducing a bifunctional ligand capable of interacting with hydroxyl and carboxyl groups of GHB. The interaction between the bifunctional ligands of the gold nanoparticles (AuNPs) and GHB triggers the aggregation of AuNPs, which induces a bathochromic shift in the surface plasmon resonance (SPR) absorption band and a visible color change from red to blue. This modification expanded the GHB detectable range to 0~35 mmol L^−1^ and enabled a visible color change from red to blue.

Son et al. [32] developed a colorimetric paper-based sensor utilizing 10,12-pentacosadiynoic acid (PCDA) nanofibers functionalized with the GHB-selective receptor gabazine. Upon drop-casting of the sample, the sensor exhibits an immediate (<1 min) color transition from blue to red in the presence of GHB, with a visual detection limit of 96 µg mL^−1^. The flexible and lightweight strip enables direct discrimination of GHB in colored and alcoholic beverages without the need for sample pretreatment. In addition, a freely available smartphone application quantifies red-channel intensity, providing an objective “safe/danger” read-out. The sensor maintains functional stability over several weeks under ambient storage conditions and can be readily mass-produced via electrospinning. These features illustrate the potential of PCDA-based materials for low-cost, on-site screening applications in the prevention of drink-spiking.

Hydrogels, characterized by their three-dimensional polymeric network, are capable of absorbing substantial amounts of water to form a colloidal system. When integrated with colorimetric sensing strategies, interactions between target analytes and the hydrogel matrix can induce distinct color changes, facilitating qualitative and quantitative detection. Ha et al. [33] developed portable hydrogel-based self-protection products (SPPs) designed to mitigate GHB-related sexual risks in social settings. The sensing platform is based on the high selectivity of 2-(3-bromo-4-hydroxystyryl)-3-ethylbenzothiazol-3-ium iodide (BHEI) toward GHB. Incorporation of BHEI into the hydrogel matrix enables specific binding with GHB, resulting in a visible color change from yellow to red. The system offers simple operation and rapid response. Moreover, GHB concentrations can be quantitatively determined using an integrated smartphone-based colorimetric detection application, achieving a detection limit of 10 mg mL^−1^, which is sufficient for rapid screening in forensic or crime scene applications.

**Figure 2:**
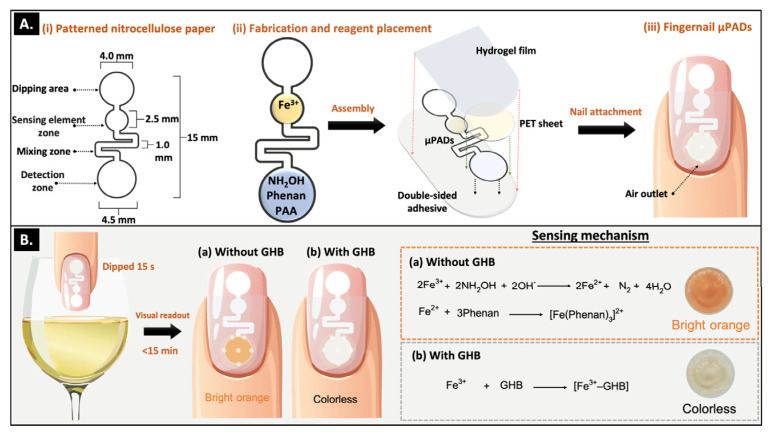
(**A**) Fingernail μPAD Development; (**B**) GHB Detection Procedure [34].

In recent years, microfluidic paper-based analytical devices (μPADs) have gained significant attention for portable chemical analysis in forensic analysis. This is due to their low fabrication cost, minimal reagent consumption, ease of use, and compatibility with capillary-driven flow. Kunpatee et al. [34] propose a wearable fingernail-based μPAD as a simple and visual screening tool for GHB detection in beverages (Figure 2). This device integrates a FeCl_3_-based colorimetric reaction system onto patterned paper fixed onto an artificial fingernail. When immersed in a beverage sample, GHB binds with Fe^3+^, inhibiting its reduction to Fe^2+^, resulting in a visible color change from orange to colorless and can be visually identified within 15 min. The sensor provides a detection limit of 0.55 μg mL^−1^ (digital analysis) and a naked-eye cutoff at 10 mg mL^−1^. This presents a practical platform for real-time, on-site GHB screening for personal safety enhancement and drug-facilitated sexual assault crime prevention in everyday social settings, see Figure 2.

**Figure 3:**
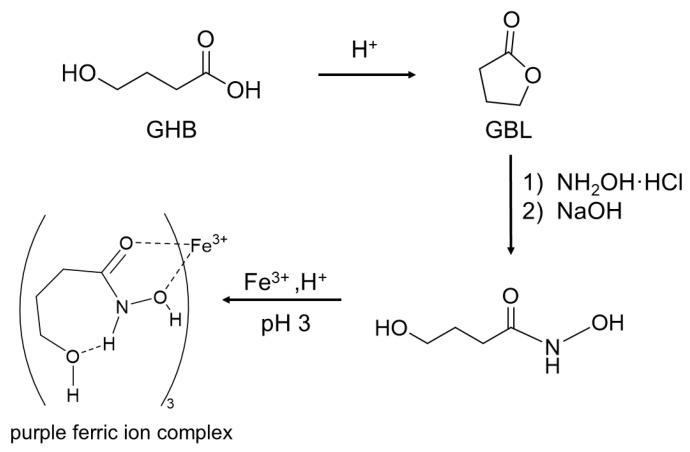
Ferric hydroxamate GHB colorimetric assay reaction sequence.

Procida et al. [35] developed a smartphone-based colorimetric method for the quantitative detection of GHB and GBL in beverages, representing the first application of colorimetry for their quantitative analysis. Under acidic conditions, GHB is converted into GBL, an organic ester that can be detected using the iron hydroxylate test. Specifically, GBL reacts with hydroxylamine hydrochloride under alkaline conditions to form γ-hydroxybutyryl hydroxylamine, which subsequently reacts with ferric chloride under acidic conditions to produce a purple-red complex (Figure 3). This complex exhibits a characteristic absorption peak at 499 nm, enabling detection in conjunction with ultraviolet/visible spectroscopy (UV/Vis). Quantitative analysis is achieved by capturing images of the color development process using a smartphone camera and extracting the red, green, and blue (RGB) color component values with a freely available application. These values are then used to construct a calibration curve for the quantification of GHB and GBL. Notably, in alcoholic beverages containing 0.56 mg mL^−1^ ethanol, the detection limit for GBL was reported to be 73.9 mg L^−1^. GHB can also be quantified using the same approach. Although these colorimetric assays are straightforward and yield results within 10 min, the primary limitation is low sensitivity, with limits of detection typically in the range of 100 to 500 μg mL^−1^.

In a recent study, Li et al. [36] introduced a novel superwettable microchip for the rapid and visual detection of GBL in beverages. The microchip incorporates Fe_3_O_4_ nanoparticles onto a superhydrophobic fabric, forming reactive sites that effectively capture microdroplets of GBL-containing samples. Upon acid treatment, Fe^3+^ ions are released and subsequently react with GBL to generate a visibly colored complex, enabling naked-eye detection. Quantitative analysis is further achieved through smartphone-based RGB imaging, providing a detection range of 0.1–10 mg mL^−1^ and demonstrating good reusability over at least 30 cycles.

### 2.2. Electrochemical Detection Methods

Electrochemical detection methods leverage the electrochemical properties of analytes for both qualitative and quantitative analyses, encompassing techniques such as cyclic voltammetry, polarography, and potentiometry [37]. These methods are lauded for their straightforward instrumentation, operational convenience, and rapid detection capabilities, which have enabled their broad application in detecting illicit food additives, pharmaceuticals, and narcotics [38,39]. GHB, characterized by a relatively simple molecular structure, possesses only hydroxyl and carboxyl functional groups that exhibit electrochemical activity. However, studies investigating its electrochemical behavior remain limited. In 2013, Jiménez-Pérez [40] conducted a pioneering study on the electrochemical reactions of GHB on platinum electrodes in slightly acidic solutions using cyclic voltammetry. The results demonstrated that the hydroxyl group of GHB can be oxidized to succinic acid under acidic conditions. The corresponding cyclic voltammograms exhibited a wide potential range for GHB, with two oxidation peaks, and the peak currents showed a concentration-dependent relationship with GHB. This work established a crucial foundation for the subsequent development of electrochemical methods for GHB detection.

Subsequently, the team synthesized platinum nanoparticles in combination with polyvinyl alcohol to modify the electrode surface, yielding a nanostructured platinum/polyvinyl alcohol-modified glassy carbon electrode. This system exploits the principle that the presence of GHB in ethanol inhibits the oxidation peak current of ethanol, enabling indirect detection of GHB in alcoholic beverages [41]. The findings demonstrated that the modified electrode exhibited high catalytic efficiency, while the presence of ethanol did not interfere with detection performance. The method achieved a limit of 0.110 mg mL^−1^, with a linear response range from 0.125 to 9.15 mg mL^−1^. Notably, the entire analytical process could be completed within 15 min.

### 2.3. Ion Mobility Spectrometry

Ion Mobility Spectrometry (IMS) is an analytical technique that detects compounds by measuring the mobility of gas-phase ions under a weak electric field. In IMS, analytes are ionized using sources such as electrospray ionization, atmospheric pressure photoionization, or radioactive isotopes (63Ni), and the resulting ions are introduced into a drift tube. Separation is achieved based on differences in ion drift times, thereby eliminating the need for chromatographic separation and significantly reducing analysis time compared to conventional methods [42]. IMS offers several advantages, including operation at atmospheric pressure, minimal sample preparation, rapid analysis, and high sensitivity. These attributes make it particularly suitable for the trace detection of volatile organic compounds. Consequently, IMS has been widely employed as a screening tool for drugs and explosives for nearly three decades [43].

Mercer et al. [44] devised an IMS-based method for the detection of GHB in urine, employing a non-split injector for direct sample introduction and thermal desorption, enabling samples to be introduced directly into the IMS instrument. Separation of the analyte from the aqueous matrix is achieved based on differences in boiling points and volatility. Notably, common potential interferents, including ethanol, methanol, acetone, isopropanol, and acetaldehyde, do not significantly affect analytical performance. The method achieves a detection limit of 3 μg mL^−1^ for GHB. Despite these advantages, IMS exhibits certain limitations in practical applications, including relatively limited selectivity and susceptibility to false positives. Furthermore, enhancing detection sensitivity remains a key objective for broadening the applicability of this technique.

### 2.4. Nuclear Magnetic Resonance

Nuclear Magnetic Resonance (NMR) technology enables direct structural elucidation of unknown toxins, making it a powerful tool for identifying new compounds without reliance on analytical standards. It is particularly effective for the rapid characterization of complex samples. In addition, NMR is inherently non-destructive and typically requires minimal sample pretreatment, eliminating the need for extraction or derivatization steps, significantly reducing the preparation time and enhancing its suitability for rapid screening of drugs [45].

The primary mode of GHB abuse involves its illicit addition to beverages and other aqueous solutions, typically in the form of carboxylate salts, most commonly sodium salts. Depending on pH conditions, GHB can exist as GHB, GHB carboxylate salts, and GBL, which are readily interconvertible and thus difficult to distinguish analytically. Therefore, beverage samples suspected of GHB contamination may in fact contain a mixture of GHB, GHB carboxylate salts, and GBL [46,47]. Most analytical methods require pH adjustment during sample pretreatment, which can alter the equilibrium among these forms. However, the absence of unified international regulations governing GHB, GHB carboxylate salts, and GBL means that such alterations in composition may introduce legal complexities, potentially complicating evidentiary interpretation and affecting conviction and sentencing outcomes.

Defrancesco et al. [48] established a detection method for GHB in aqueous solutions using ^1^H nuclear magnetic resonance (^1^H NMR). This approach only requires simple dilution of the sample with deuterium oxide (D_2_O) without pH adjustment or additional pretreatment. This approach minimizes potential inaccuracies associated with the interconversion of GBL to GHB during analysis, offering a rapid and convenient method for the direct identification and quantification of GHB in aqueous media. Moreover, ^1^H NMR has been effectively utilized in the detection of GHB and GBL in human biological specimens, including urine, serum, and saliva, demonstrating high sensitivity and broad applicability [49,50].

### 2.5. Fluorescence Method

Fluorescence detection offers higher sensitivity than traditional colorimetry and provides a more objective interpretation of analytical results. By designing fluorescence probes tailored to the chemical characteristics of target compounds, this method enables the analysis of complex samples without the need for pretreatment, thereby facilitating real-time analysis with operational simplicity [51]. Although studies on fluorescence-based detection of GHB remain limited, the general strategy involves the development of specialized fluorescent probes. Variations in substituent groups and their electronic properties can significantly influence the charge distribution within the probe molecules. This allows GHB to interact with probes from multiple orientations, where recognition is mediated through non-covalent interactions such as hydrogen bonding and van der Waals forces, ultimately producing distinguishable optical responses. Rodríguez-Nuévalos et al. [52] and Baumes et al. [30] successfully achieved both quantitative and qualitative GHB detection by leveraging hydrogen-bonding interactions and ligand-exchange mechanisms. Hernández-Contreras et al. [53] developed an eco-friendly cellulose paper-based fluorescent probe for the real-time detection of GHB in beverages. The sensor utilizes a 2-aminonaphtoxazole fluorophore and exhibits a visible fluorescence enhancement under UV light, with a detection limit of 7.3 mM. This platform is suitable for on-site screening applications aimed at preventing drink spiking.

Garrido et al. [54] reported a lateral-flow strip that integrates a coumarin-343/Cu^2+^ indicator-displacement assay on a PEG-coated glass-fiber membrane. In this system, GHB displaces the dye from the Cu^2+^ complex, leading to the restoration of fluorescence and enabling smartphone-based quantification of the analyte in soft drinks and alcoholic beverages within 1 min and with a detection limit of 0.1 µM.

Ryu and Kim [55] reported a BODIPY-metal complex (**1·Fe^3+^**) as a simple, rapid, and sensitive colorimetric and fluorimetric indicator for the detection and quantification of GHB. This sensing mechanism is based on ligand displacement, whereby GHB competitively binds to the Fe^3+^ center, triggering a visible color change accompanied by a fluorescence “turn-on” response. The assay demonstrated a low detection limit of 0.01 μg mL^−1^ (fluorescence), rapid response time (<5 s), and compatibility with complex beverage matrices, including both alcoholic and non-alcoholic. This approach offers exceptional sensitivity and speed, though it relies on metal ion complexation and may require careful pH control. Rodríguez-Nuévalos et al. [56] also reported a hydrazone-BODIPY platform that operates via pH-induced deprotonation of the probe upon interaction with GHB, yielding a slightly higher LOD of 0.3 μM and enabling dual detection of both GHB and synthetic cathinones, thus expanding its applicability in multi-drug screening scenarios, albeit with a more complex probe design. Both methods rely on colorimetric and fluorescent dual-mode readouts for on-site visual detection, providing rapid, low-cost alternatives to conventional laboratory techniques.

Furthermore, Moss et al. [57] recently developed a fluorescent ionic liquid nanosensor for the onsite detection of GHB based on trihexyltetradecylphosphonium fluorescein (THP_2_FL). The as-prepared nanoparticles with a uniform size of 199 nm exhibited a remarkable fluorescence enhancement (up to 60%) and absorbance increase (79%) upon exposure to GHB, enabling rapid and visual recognition. This ionic liquid system shows unique superiority including simple synthesis, low cost, good water solubility and high stability although it has a higher LOD with 10 μg mL^−1^. And it should be noted that 1,4-BD and ethanol may cause signal interference.

**Figure 4:**
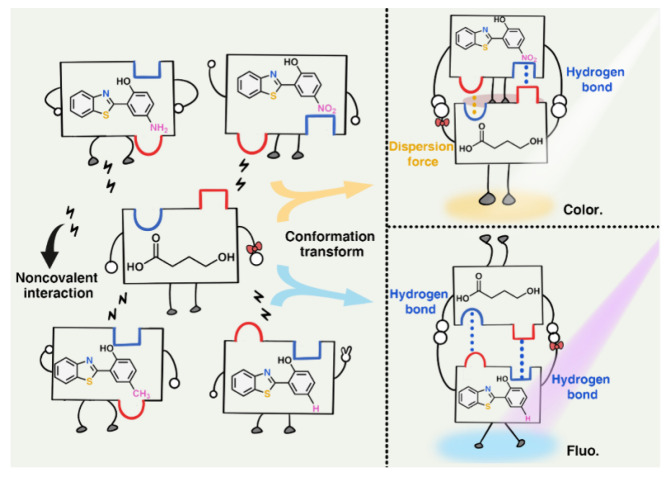
Principle of HBT-based probe recognition of GHB molecules [58].

**Figure 5:**
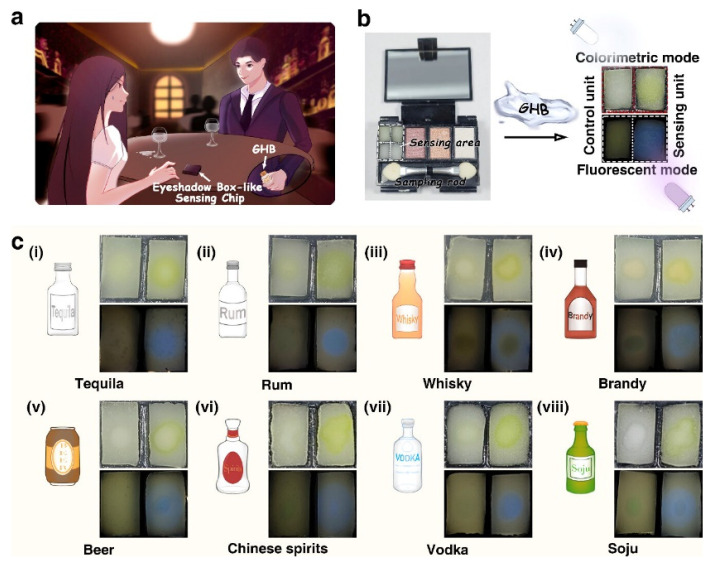
Schematic illustration of the portable eyeshadow box sensor. (**a**) Application scenario. (**b**) Internal structure of the sensor. After the brush was exposed to suspected beverages containing GHB and brought into contact with the sensing units, visible yellow coloration and blue fluorescence were observed under natural light and 365 nm UV illumination. (**c**) Images of the sensor used for detecting GHB adulteration in eight common alcoholic beverages: (**i**) Tequila, (**ii**) Rum, (**iii**) Whisky, (**iv**) Brandy, (**v**) Beer, (**vi**) Chinese spirits, (**vii**) Vodka, and (**viii**) Soju. Characteristic yellow coloration and blue fluorescence were observed exclusively in GHB-positive samples, demonstrating high specificity in complex alcoholic beverage matrices [58].

Recently, Zhang et al. [58] proposed an electronic-effect-driven recognition strategy for the visual detection of GHB. They designed and synthesized four HBT-derived fluorescence probes (HBT-NO_2_, HBT-H, HBT-CH_3_, and HBT-NH_2_), achieving highly sensitive, highly specific, rapid, and dual-mode (colorimetric-fluorescent) visual detection of GHB. Notably, the HBT-NO_2_ and HBT-H probes exhibit a yellow colorimetric response and a blue fluorescent signal upon interaction with GHB, forming a dual-probe sensing system that integrates both colorimetric and fluorescence readouts. Figure 4 illustrates four HBT probes with different substituents: –NH_2_ (electron donating), –CH_3_ (weak electron donating), –H (neutral), and –NO_2_ (electron withdrawing). GHB possesses a relatively simple molecular structure with weak chemical activity, making its detection challenging using conventional covalent or hydrogen-bonding synergy. By exploiting the electronic effects of different substituents, the probes exhibit distinct optical responses upon interaction with GHB. When the HBT–NO_2_ probe interacts with GHB, the electron withdrawing nitro group alters the electron distribution within the probe. This interaction narrows the HOMO LUMO energy gap, causing a red shift in the absorption spectrum. Consequently, the solution changes from colorless to a bright yellow color with an absorption peak at 416 nm, thereby providing a clear colorimetric signal. In contrast, when the HBT–H probe containing a neutral substituent bind to GHB, a different sensing mechanism is observed. GHB binds more strongly than the solvent to the hydroxyl oxygen of the probe, promoting an enol to ketone tautomerization. This transformation generates a new strong emission peak at 472 nm, producing a “turn-on” blue fluorescence under 365 nm UV irradiation. Thus, through the electronic effect driven modulation, the –NO_2_ probe enables colorimetric detection, whereas the –H probe enables fluorescent detection, together achieving rapid, sensitive, and specific recognition of GHB. The sensing system achieves an exceptionally low-detection limit of 0.586 ng mL^−1^ and a rapid response time of approximately 0.2 s. Importantly, structural related analogs such as 1,4-BD, n-butanol, and lactic acid, as well as common food-related substances, including xylitol, tea polyphenols, and glucose, do not interfere with GHB detection. Moreover, to address the needs of preventive and covert detection for potential victims, the team developed a portable, eyeshadow box-style sensor. This innovation enables discreet, accurate, and visual detection of illicit GHB in 13 common types of alcoholic and non-alcoholic beverages (Figure 5).

Recently, Hernández Contreras et al. [59] further advanced optical sensing strategies for the detection of GHB in complex biological matrices. They developed two silica-based optical sensors (S1 and S2) functionalized with 2 aminonaphtoxazole derivatives for rapid, on-site GHB detection. Among these, the chromogenic sensor S1 enabled naked eye colorimetric recognition with a detection limit of 2.21 μM, whereas the fluorogenic sensor S2 achieved a lower LOD of 1.65 μM under UV light excitation, with both responses occurring instantaneously within seconds. Moreover, a precursor sensor S1 was successfully applied for the visual detection of GHB in oral fluid samples, displaying a distinct pink red color change and a LOD of 19.2 μM within a linear range of 32–132 μM. Collectively, these solid phase and solution phase optical sensors provide rapid, sensitive, and portable platforms for GHB detection in both beverages and saliva, thereby substantially broadening the application scope of colorimetric and fluorescent detection methods.

Despite the escalating misuse of GBL as a date-rape prodrug, reports on rapid, on-site testing remain scarce. In 2013, Zhai et al. [60] developed the first fluorescent sensor for GBL. A 3,5-dihydroxyl compound (named **Green Date**) was identified as an effective probe, exhibiting a strong fluorescence response to GBL. The detection process is completed within a few seconds, with a detection limit of 3 mg mL^−1^ in aqueous solution. The sensor was subsequently demonstrated to function across a range of pH conditions and in the presence of 10% ethanol. It is also capable of detecting GBL in various beverage matrices, producing a distinct and obvious color change. This discovery represents a significant advancement in strategies aimed at mitigating drug-facilitated sexual assault.

### 2.6. Capillary Electrophoresis

Capillary electrophoresis (CE) has emerged as a powerful technique for the separation and analysis of a wide range of compounds, spanning small inorganic ions to large biomolecules, such as proteins and DNA [61]. Among its various modes, capillary zone electrophoresis (CZE) and micellar electrokinetic chromatography (MEKC) are most commonly employed for the analysis of abused substances. In 1995, Altria and Howells [62] developed a MEKC method for the analysis of organic solvents, using a carrier electrolyte comprising sodium dodecyl sulphate (SDS) and barbital as a UV-absorbing agent to allow indirect UV detection. Dahlén and Vriesman [63], as well as Bishop et al. [64], applied CE for the determination of GHB in seized drug samples and beverages using direct UV-detection. However, due to the inherently weak UV absorbance of the compound, the methods exhibited relatively high limits of detection.

In 2004, Gottardo et al. [65] applied CZE with indirect detection for the quantitative determination of GHB in human urine. They subsequently developed a rapid CZE-based method for the direct determination of GHB in human urine and serum at potentially toxic concentrations [66]. In this approach, sample pretreatment was simplified to a 1:8 dilution with 3 mM NaOH, and calibration curves prepared in water, urine, and serum exhibited good linearity over the concentration range of 25–500 μg mL^−1^. Baldacci et al. [67] also demonstrated the determination of GHB in biological fluids. However, their approach required liquid–liquid extraction of urine samples prior to analysis, which reduces operational convenience.

Contactless conductivity detection, commonly referred to as capacitively coupled contactless conductivity detection (CE-C^4^D), represents an alternative detection method for CE. A key advantage of CE-C^4^D is its potential for instrument miniaturization, enabling portable systems suitable for on-site or forensic applications. Gong et al. [68] were the first to develop a rapid analytical method based on CE-C^4^D for the determination of GHB in clinical samples. The method achieved a detection limit of approximately 2 μg mL^−1^ in clinical matrices, which is sufficiently sensitive for identifying GHB overdosage and adequate for measuring endogenous concentrations in urine.

Recently, saliva has emerged as an alternative biological matrix to urine and blood for forensic drug testing, as the collection of urine or blood samples typically requires specialized procedures and facilities. In contrast, saliva sampling is non-invasive, rapid, and can be easily performed on-site by law enforcement personnel. Moreover, saliva is a promising analytical matrix containing a wide range of compounds that may serve as biomarkers for health disorders [69]. CE-C^4^D has been successfully applied to the analysis of simple organic and inorganic compounds in saliva by Kubáň et al. [70] In 2015, Mazina et al. [71] first implemented CE-C^4^D with sequential indirect UV detection for the determination of GHB in saliva samples. Validation results showed that CE-C^4^D provides sufficient sensitivity to distinguish between endogenous and exogenous concentrations of GHB in saliva samples. The method exhibited instrument detection and quantification limits of 0.49 and 1.6 mg L^−1^ for C^4^D, respectively, and 5.1 mg L^−1^ and 17.0 mg L^−1^ for indirect UV, respectively. Good linearity was achieved over concentration ranges of 2.5–400 mg L^−1^ for C^4^D and 12.5–400 mg L^−1^ for indirect UV detection. The proposed methodology offers several key advantages, including short analysis time, high sensitivity, and simple sample preparation. Furthermore, it is amenable to automation and miniaturization, highlighting its potential for on-site confirmatory analysis. Saar-Reismaa et al. [72] further demonstrated the good applicability of capillary electrophoresis coupled with a CE-C^4^D for the determination of GHB in saliva, with the limits of detection and quantification for GHB in saliva being 1.5 and 5.0 mg L^−1^, respectively.

Compared with other separation methods, CE offers significant advantages in terms of miniaturization, enabling the development of portable instruments suitable for on-site analysis. Růžička et al. [73] developed a compact and cost-effective sampling device integrated with portable CE to evaluate GHB in simulated saliva samples. Although slight non-linearity was observed at higher concentrations (>10 mg L ^−1^), the method achieved a limit of detection of approximately 1.9 mg L^−1^, which is adequate for on-site forensic investigations.

### 2.7. Raman Spectroscopy

Raman spectroscopy enables drug detection by analyzing the inelastic-scattering fingerprints of molecular bonds. Its non-destructive, rapid analysis (within seconds), ability to penetrate containers, and compatibility with portable instrumentation make it ideal for on-site screening applications. Brewster et al. [74] demonstrated that both GHB and its pro-drug GBL can be non-destructively identified in beverages at concentrations as low as 1% *w*/*v* (GHB) and 0.25% *v*/*v* (GBL) using 785 nm Raman spectroscopy. Notably, both hand-held and bench-top instruments produced comparable results, allowing rapid (15 s) screening of suspected drinks without the need to open the container.

Munshi et al. [47] further extended this approach to monitor GHB-GBL inter-conversion, using the same portable Raman-in-a-suitcase (RIAS) to track equilibrium dynamics in real beverage matrices (pH 2–7, 5–40 °C) for 52 days. Their results indicated that significant conversion occurs only under conditions of pH ≤ 4 and temperatures ≥ 23 °C. Importantly, the characteristic Raman signatures of both compounds remain sufficiently stable during the initial hours following spiking, enabling reliable identification.

Collectively, these studies demonstrate that hand-held 785 nm Raman systems can rapidly (within seconds) detect GHB-GBL through sealed bottles, with detection limits well within the range of intoxicating doses. In addition, these systems allow real-time monitoring of the acid-catalyzed lactonization processes that may occur in drinks. Overall, the technique represents a mature proof-of-concept for field applications. However, current studies are largely limited to clear or lightly colored liquids. Strongly fluorescent beverages, such as cola and red wine, and quantitative resolution of GHB/GBL mixtures, remain significant challenges.

## 3. Conclusions

GHB, a relatively recent psychoactive substance, has been increasingly abused, often through illicit addition to beverages or involvement in drug-facilitated sexual assault.

Its synthetic precursors, GBL and 1,4-BD, are also clandestinely mixed into beverages as GHB substitutes and can be rapidly metabolized into GHB in the human body, posing significant risks to public safety. The rapid absorption and metabolism of GHB, along with its short detection window, necessitate the development of rapid and sensitive detection techniques for effective screening. However, the interconversion between GBL and GHB during analytical procedures may alter the original composition of the sample. In addition, the endogenous presence of GHB in the human biological matrices further complicates the interpretation of analytical results, thereby posing additional challenges to the development and application of rapid detection technologies for GHB and its precursors.

This review summarizes various rapid detection technologies for GHB and GBL, including colorimetric methods, electrochemical analysis, ion mobility spectrometry, nuclear magnetic resonance, fluorescence-based methods, and capillary electrophoresis, applied to matrices such as beverages and human biological samples (Table 1). Colorimetric approaches are generally low-cost and user-friendly, and portable platforms such as paper-based sensors and wearable microfluidic chips require minimal reagents and no specialized training, making them suitable for large-scale on-site screening, despite relatively low sensitivity and specificity. Electrochemical sensors offer simple instrumentation and straightforward manipulation; however, their performance is restricted by the weak electrochemical activity of GHB, necessitating advanced electrode modification. Ion mobility spectrometry enables rapid detection but is limited by poor anti-interference capability, high susceptibility to environmental conditions, and relatively high false-positive rates. Nuclear magnetic resonance effectively avoids interconversion interference among GHB analogues; however, its exorbitant instrumental and maintenance costs significantly limit field applicability. Fluorescence-based sensing provides ultrahigh sensitivity, rapid response, and excellent portability, and smartphone-assisted quantification further improves accessibility; nevertheless, the simple molecular structure of GHB restricts probe diversity, and research in this area remains relatively underdeveloped. Capillary electrophoresis exhibits prominent separation performance for small ionic analytes. Miniaturized CE systems require minimal sample pretreatment and show satisfactory anti-interference capacity for saliva, urine, and serum samples, although their performance may become unstable under fluctuating temperature and buffer conditions. Raman spectroscopy allows non-destructive, container-penetrating, and ultrafast in situ detection, offering significant forensic potential; however, its application is hindered by fluorescence background interference from dark-colored beverages and insufficient quantitative resolution for mixed samples.

With regard to real-world reliability and robustness, most validated rapid detection methods demonstrate favorable tolerance to complex matrices, including alcoholic beverages, non-alcoholic drinks, and human biofluids. Modified electrochemical sensors can effectively eliminate ethanol interference in alcoholic matrices, while dual-channel fluorescent–colorimetric probes effectively resist structural analogues and common food additives. Certain portable sensing platforms exhibit excellent reusability; for example, superwettable microchips can sustain more than 30 detection cycles. Nevertheless, several inherent limitations still constrain field deployment. Temperature fluctuations can reduce the analytical precision of capillary electrophoresis, while variable ambient conditions increase the false-positive probability of ion mobility spectrometry. Therefore, further optimization strategies, such as the integration of embedded temperature control modules and the application of algorithm-based calibration, are recommended to enhance environmental adaptability.

The limited number of chemically active functional groups and the relatively simple linear structure of GHB also restrict the diversity of feasible fluorescence probes. Given the distinctive physicochemical properties of GHB and its precursors, there is an urgent need to further optimize and refine existing rapid detection technologies.

To effectively curb substance abuse, prevent and respond to drug-facilitated sexual assault, maintain personal safety and social stability, and meet the requirements for on-site forensic investigations, the development of sensitive, precise, and portable rapid detection platforms is essential.

## Figures and Tables

**Table 1:** Overview of LOD for rapid detection of GHB.

Method	Matrix	LOD	Reference
Colorimetry	beverage	5 × 10^5^~2 × 10^6^ ng mL^−1^	[24]
	urine	5 × 10^5^ ng mL^−1^	
	beverage	4 mmol L^−1^	[30]
	beverage	1.12 mmol L^−1^	[31]
	beverage	9.6 × 10^4^ ng mL^−1^	[32]
	beverage	10 mg mL^−1^	[33]
	beverage	0.55 μg mL^−1^	[34]
	beverage	7.39 × 10^4^ ng mL^−1^	[35]
	beverage	10^5^ ng mL^−1^	[36]
Electrochemical detection	beverage	1.1 × 10^5^ ng mL^−1^	[41]
Ion Mobility Spectrometry	urine	3000 ng mL^−1^	[44]
Nuclear Magnetic Resonance	beverage	/	[49]
	saliva	/	[50]
Fluorescence	beverage	7.3 mmol L^−1^	[53]
	beverage	10^−4^ mmol L^−1^	[54]
	beverage	10 ng mL^−1^	[55]
	beverage	3 × 10^−4^ mmol L^−1^	[56]
	beverage	10 μg mL^−1^	[57]
	beverage	0.586 ng mL^−1^	[58]
	saliva	0.0192 mmol/L	[59]
Capillary electrophoresis	solution	5.1 × 10^3^ ng mL^−1^	[63]
	urine, serum	2.5 × 10^4^ ng mL^−1^	[66]
	urine, serum	2 × 10^3^ ng mL^−1^	[68]
	saliva	5.1 × 10^3^ ng mL^−1^	[71]
	saliva	1.5 × 10^3^ ng mL^−1^	[72]
	saliva	1.9 ×10^3^ ng mL^−1^	[73]
Raman spectroscopy	beverage	1% *w*/*v*	[74]

## Data Availability

No new data were created or analyzed in this study.
